# Subsidized Sachet Water to Reduce Diarrheal Disease in Young Children: A Feasibility Study in Accra, Ghana

**DOI:** 10.4269/ajtmh.15-0854

**Published:** 2016-07-06

**Authors:** James Wright, Mawuli Dzodzomenyo, Günther Fink, Nicola A. Wardrop, Genevieve C. Aryeetey, Richard M. Adanu, Allan G. Hill

**Affiliations:** ^1^ Geography and Environment, University of Southampton, Southampton, United Kingdom.; ^2^ Ghana School of Public Health, University of Ghana, Legon, Ghana.; ^3^ Department of Global Health and Population, School of Public Health, Harvard University, Boston, Massachusetts.; ^4^ Social Sciences, University of Southampton, Southampton, United Kingdom.

## Abstract

Use of drinking water sold in plastic bags (sachet water) is growing rapidly in west Africa. The impact on water consumption and child health remains unclear, and a debate on the taxation and regulation of sachet water is ongoing. This study assessed the feasibility of providing subsidized sachet water to low-income urban households in Accra and measured the resultant changes in water consumption. A total of 86 children, 6–36 months of age in neighborhoods lacking indoor piped water, were randomized to three study arms. The control group received education about diarrhea. The second arm received vouchers for 15 L/week/child of free water sachets (value: $0.63/week) plus education. The third arm received vouchers for the same water sachet volume at half price plus education. Water consumption was measured at baseline and followed for 4 months thereafter. At baseline, 66 of 81 children (82%) drank only sachet water. When given one voucher/child/week, households redeemed an average 0.94 vouchers/week/child in the free-sachet-voucher arm and 0.82 vouchers/week/child in the half-price arm. No change in water consumption was observed in the half-price arm, although the study was not powered to detect such differences. In the free-sachet-voucher arm, estimated sachet water consumption increased by 0.27 L/child/day (*P* = 0.03). The increase in sachet water consumption by children in the free-sachet-voucher arm shows that provision of fully subsidized water sachets might improve the quality of drinking water consumed by children. Further research is needed to quantify this and any related child health impacts.

## Introduction

Household use of water sold in plastic bags, typically having a volume of 500 mL, (sachet water) has been expanding rapidly in west Africa since they first became available from water vendors in the late 1990s.^1^ The growth of this industry reflects the global growth in packaged water, with 225 billion liters of bottled water consumed worldwide in 2011.^2^ In urban Ghana, the share of households using sachet water as the main source of drinking water expanded from 5.7% in 2003 to 37.0% in 2008.^1^ Rates of sachet water consumption have remained lower in poorer households, and there is some evidence that consumption of sachet water is linked to rationing of the piped water supply.^3^,^4^ As with water access patterns more generally,^5^ understanding of sachet water consumption patterns is based on cross-sectional data, with very little information available on consumption patterns and differences among households.

Recent evidence suggests that sachet water in Ghana is relatively clean, with low average fecal bacteria counts. A small study found 60 sachet water samples from a low-income community in Accra^6^ to be compliant with national regulations for indicator bacteria.^7^ Similarly, a recent nationally representative household survey, the Ghana Living Standards Survey Round 6, found lower rates of detectable *Escherichia coli* in sachet water samples at the point of consumption than in samples taken from piped systems.^8^ This may reflect a combination of water treatment by prefiltration, reverse osmosis, and/or ultraviolet prior to packaging^9^ and the protective effect of packaging against the recontamination typically associated with stored water.^10^ Unlike bottles, sachets cannot be reused, thereby avoiding potential contamination associated with refilling. Sachet water use has been found to be associated with higher self-reported overall health in women, and lower likelihood of diarrhea in children.^3^

Policy responses to the emergence of this industry have varied. In Ghana, regulation of sachet manufacturing is the responsibility of the Food and Drugs Authority (FDA) and the Ghana Standards Authority (GSA).^1^ Sachet production and/or distribution by unregulated small-scale producers and wholesalers is a public health concern, whereas discarded sachet plastic sleeves have become a major environmental issue.^1^ A public–private partnership has established a waste management system in Accra, whereby waste collectors pay households for sachet “rubbers” (bundles of used plastic sleeves) and then sell them to recycling companies. Ongoing concerns of waste and regulation have resulted in plans for a “gradual” ban on sachet water in countries such as Nigeria,^9^ whereas a tax to fund the management and recycling of sachet plastic waste was proposed and then rescinded in Ghana.^1^ In 2013, sachet water became subject to value added tax (at 17.5%, including a 2.5% National Health Insurance levy) under Schedule 1 of the Value Added Tax Act. This tax, in addition to the excise duty on sachet sleeve plastic which is currently in place in Ghana and elsewhere, affects the affordability of sachet water.

The objective of this study was to evaluate the feasibility of providing subsidized sachet water to low-income households in Accra through a voucher system and to assess its impact on child and household water consumption patterns. The long-term study objective was to provide data supporting a subsequent, large-scale trial of the impact of subsidized sachet water on child health, thereby assisting the formulation of policies relating to sachet water use as described above.

## MATERIALS AND METHODS

### Trial design.

In this parallel-group trial, participants were randomly allocated to a control arm, a partially subsidized sachet water arm, and a fully subsidized sachet water arm. Households were not blinded to study arm allocation. The study received ethical approval from the Noguchi Memorial Institute for Medical Research in Ghana (ref: 006/14-15) and from the University of Southampton in the United Kingdom (ref: 12241).

### Eligibility criteria.

Children were recruited in eligible enumeration areas (EAs) where, according to data from the 2010 census, a majority of households were without running water piped into the home. Eligible households included at least one adult and at least one child 6–36 months of age.

### Setting.

Four EAs in two high population density, low-income neighborhoods of Accra, Ghana, were chosen for the study. Census statistics showed that sachet water was the predominant drinking water source in two EAs, whereas in the other two EAs, drinking water was obtained either from standpipes or was piped to the yard. In all four EAs, water for cooking and hygiene was predominantly from standpipes or outdoor taps.

### Interventions.

The primary intervention was distribution of vouchers for bags of sachet water every 2 weeks. Households in the full-subsidy arm received vouchers for 15 L of sachet water (30 × 0.5 L bags) per week for each eligible child for a period of 4 months. The value of each 15-L voucher was US$0.63. Households in the partially subsidized arm received vouchers to obtain the same weekly volume of sachet water but at a 50% discount. The water quantity used for the intervention was based on daily recommendations for fluid intake in young children. The World Health Organization recommends a daily liquid intake of 1 L/day for children weighing 10 kg and 0.75 L/day for children weighing 5 kg.^11^ In the United States, the recommendation is a liquid intake of 0.8 L/day, including 0.6 L/day of beverages (e.g., breast milk, juice, or water), for children 7–12 months of age, and 1.3 L/day for children 1–3 years of age, including 0.9 L/day of beverages.^12^ The water quantity provided in the intervention exceeded those recommendations to allow for increased liquid consumption due to the hot climate and some consumption by other household members.

In both subsidy arms, the children's caregivers (a household member aged 18 or over, with primary caring responsibilities for the child[ren]) were instructed to make sure children drank exclusively sachet or bottled water. Sachet water was distributed via existing markets: local stores and kiosks were given a small initial payment and then reimbursed for the vouchers collected, plus a 10% commission. Before the start of the intervention, a sample of 34 sachets were collected from vendors in the study area, transported on ice to a laboratory, and tested for total coliforms, *E. coli*, and total heterotrophic bacteria. All 34 samples were compliant with national regulations for these parameters.^7^ In the first 4 weeks of follow-up, households were asked to retain any used sachet sleeves from vouchers for the research team to collect. Thereafter, because it transpired, there was a waste collection system in both neighborhoods that paid for bundles of used sachets, the research team simply asked to see the used sachets.

The caregivers of all enrolled children received educational messages on diarrhea symptoms, prevention, and management in the first 4 weeks of the intervention, in accordance with the country health services guidelines.^13^ The messages were sent by short message service (SMS) to cell phones, and were read aloud if the SMS had not been read by the intended recipient. Follow-up quizzes were used to reinforce these messages.

### Sample size.

No sample size calculations were carried out because the trial was primarily intended as a feasibility study. A sample of 86 children in 80 households from four EAs was chosen to ascertain whether a voucher system would work in principle. The trial was to be stopped if any new evidence come to light via the regulators (the GSA and FDA) suggesting that the water in the sachets was unsafe.

### Household selection, randomization, and baseline survey.

After listing of all households in the study area, households in each EA were selected by systematic random sampling. Every second eligible household was selected in each location to maximize distances between participating households and thereby minimize contact between households in different arms. Informed, written consent was obtained from selected adult children's caregivers prior to participation in the study. A global quota was used to balance the number of households randomly assigned to each arm, and household selection and allocation to the three study arms was conducted using a computer-generated list of random numbers. An intervention field team under the direction of a trial manager enrolled the participants and monitoring was carried out by a team that was not informed of the study allocations. Prior to randomization, baseline household and child characteristics were obtained using a questionnaire.

### Outcomes and follow-up monitoring.

An independent monitoring team administered questionnaires to the children's caregivers on weeks 1, 2, 3, 4, 7, 9, 11, and 12 of the intervention to collect information on water sources and consumption and child health. Instances of diarrhea were monitored by the caregivers and recorded in pictorial diaries, but the data are not analyzed here because of concerns over self-reported health outcomes in unblinded drinking water trials^14^ and adaptations needed to these pilot instruments following this exercise.

### Intervention evaluation.

A separate evaluation team conducted questionnaire-based interviews with children's caregivers in the free- and half-price sachet intervention arms to collect data on voucher receipt and redemption and sachet water use. This team also observed the sachets stored in the household and asked to see the used sachet sleeves. Their household visits were interspersed with the monitoring team visits. When the intervention had concluded, focus groups were conducted to obtain qualitative information on participant experience with the sachet vouchers and their reactions to the study in general. Four focus groups of 8–10 people each comprised a total of 34 participants from the free- or half-price sachet study arms.

### Data analysis.

The analysis focused on water sources, water consumption patterns, and voucher redemption. In addition to baseline household and demographic characteristics, summary statistics of the volume of water consumed by children and the percentage of children consuming exclusively sachet or bottled water at baseline were calculated. The χ^2^ test, Fisher's exact test, and one-way analysis of variance (ANOVA) were used to assess the statistical significance of differences between intervention arms in percentages, percentages of small cell counts and means, respectively. Univariable linear regression models were used to assess correlations of age, gender, and breastfeeding status with the total volume of water and other liquids consumed, such as infant formula, on the day prior to the questionnaire, i.e., at baseline. Models including age as continuous and categorical (3 and 6 months) variables were compared with assess the linearity of relationships with the volume of liquid consumed. The variable which resulted in the smallest Akaike information criterion (AIC) value was selected as the best fitting model.

To assess intervention delivery, we calculated the percentage of households that had not received the vouchers that they were entitled to in one or more of the intervention weeks. We also calculated summary statistics for the number of vouchers redeemed per child per intervention week, for each intervention arm. We calculated the percentage of caregivers who reported drinking sachet water obtained with intervention vouchers, and the percentage reporting that some of this sachet water was consumed by household members other than the children enrolled in the study. Direct observations were used to calculate the average number of water sachets stored within the households in the intervention arms.

The monitoring questionnaires collected information on the total water volume and the number of water sachets given to children on the previous day. The difference in total water consumption and sachet water consumption identified children who did not exclusively consume sachet water, and allowed calculation of the percentage of children exclusively consuming sachet water each week in each study arm. Summary statistics of the volumes of sachet water given to children on the day prior to the monitoring team visit were calculated in each intervention arm and reported in histograms. Summary statistics were also calculated for the volume of sachet water (not only sachets obtained with intervention vouchers) consumed by primary caregivers.

The unadjusted effect of intervention arm on the volume of sachet water given to children (based on responses to the monitoring questionnaire) was assessed using linear mixed-effects regression with a panel specification. This included random intercepts to account for clustering of observations at the individual level (i.e., repeated measures over time). An adjusted model accounting for the effects of confounders was also fitted to the data. Model comparison was used to select the most appropriate variables for adjustment, with age as a continuous and a categorical (3 and 6 months) variable, gender, baseline water and total liquid consumption, and breastfeeding status (at the time of each monitoring questionnaire), due to colinearity between variables. The variables that resulted in the largest *R*^2^ value were selected for inclusion in the final adjusted model. Statistics other than the panel regression were computed using the R statistical software package (R Foundation for Statistical Computer, Vienna, Austria).^15^ Panel regression was conducted using Stata (StataCorp, College Station, TX).^16^

## RESULTS

### Participant flow.

A total of 86 children in 80 households were enrolled and randomly assigned to control (29 children in 26 households), subsidized sachets (29 children in 27 households), and free-sachets (28 children in 27 households) arms. All households completed the study and 86 children received the intended treatment and were included in the analysis. On the delivery side, one of 16 sachet vendors (6%) dropped out toward the end of the study because study participants were not purchasing sachets from their outlet. Baseline data were not recorded for one child who was temporarily absent from the household. Recruitment began on February 16 and was completed on February 23, 2015. The intervention and follow-up were conducted for 12 weeks beginning on February 16, for follow-up of 83 days for 43 children, 82 days for 36 children, 80 days for 6 children, and 76 days for 1 child.

### Baseline characteristics.

The majority of households (87%) used sachet water as their main source of drinking water at baseline. This did not vary substantially between the three study arms (Table 1 , Fisher's exact test, *P* = 0.82). The remaining households reported various forms of piped water as the main source of drinking water. The sources of water for cooking were more diverse (Table 1). The most frequent was water from tanker trucks (30%), followed by public taps and standpipes (25%), and water from a neighbor's tap (24%). There were no statistically significant differences between intervention arms (Fisher's exact test, *P* = 0.15). However, the differences between water sources for the purposes of drinking and cooking were statistically significant (Fisher's exact test, *P* = 0.002). Most households (81%) stored drinking water at home in sachets; 11% stored drinking water storage in a covered container, and 8% did not store drinking water at home. Again, there were no statistically significant differences between arms (Fisher's exact test, *P* = 0.99).

Approximately half of the children (46%) were male (Table 1), with no statistically significant differences between intervention arms (52% in the control arm, 43% in the half-price sachet arm, and 44% in the free-sachet arm; χ^2^ test, *P* = 0.77; see Table 1). The children were between 6 and 36 months of age, and the mean age was 19.7 months, with no significant differences between intervention arms (ANOVA, *P* = 0.96). The mean volume of water consumed from any source at baseline (i.e., the day before the questionnaire was administered) was 0.89 L/day/child. This was slightly higher in the free-sachet intervention arm than the control or half-price sachet arms, but the difference was not statistically significant (Table 1; ANOVA, *P* = 0.63).

The results indicate a high level of sachet water consumption at baseline and throughout the intervention. At baseline, 66 children (81%) consumed water exclusively from sachets or bottles on the day prior to the survey, nine (11%) consumed water from other sources in addition to sachets or bottles, and five (6%) only consumed water from other sources (note that data were missing for five children, and one child reported consuming no water).

**Figure 1.**
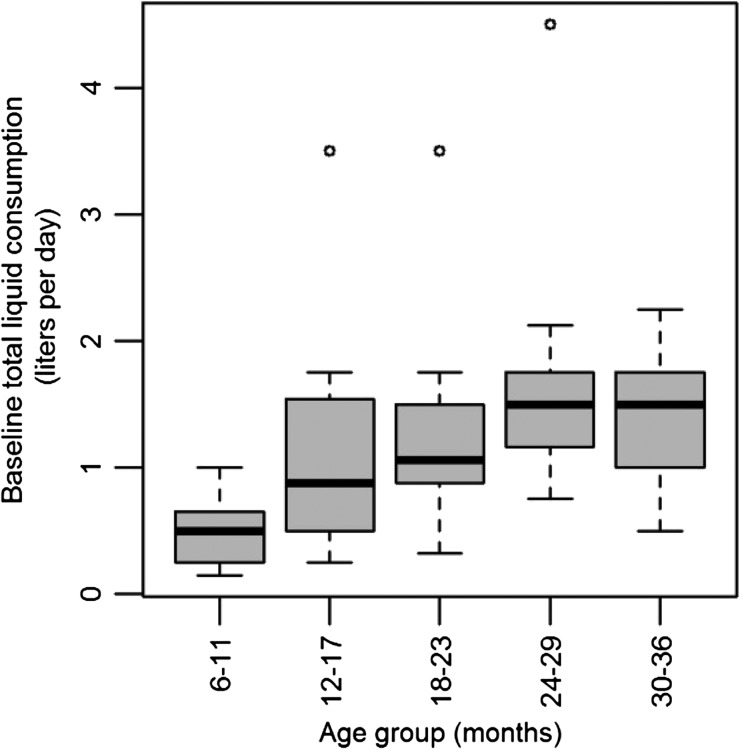
Boxplot of baseline total liquid consumption in liter/day of water and other liquids by each age group on the day prior to the baseline survey.

Age was significantly correlated with total liquid consumption (L/day) including water and other liquids, but excluding breast milk. As shown in Figure 1

, the daily volumes consumed increased with age. Univariable linear regression (Table 2 ) resulted in a smaller AIC value when age was included as a continuous variable than when it was included as a categorical variable at 3 and 6 months of age (coefficient = 0.038, *P* < 0.001). Breastfeeding status was also significantly correlated with total liquid consumption, with smaller volumes consumed in children being breastfed at baseline (coefficient = −0.85, *P* < 0.001; Table 2). Breastfeeding status was found to be the best fitting model for total liquid consumption at baseline. Gender was not significantly correlated with liquid consumption (*P* = 0.51).

### Assessment of intervention delivery.

**Figure 2.**
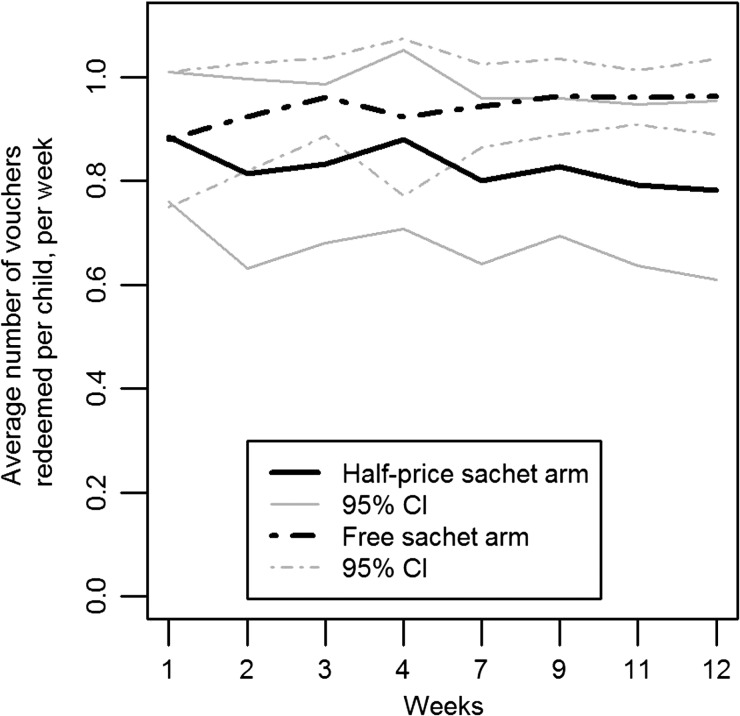
Average number of sachet water bag vouchers redeemed per child per week over the intervention period. CI = confidence interval.

The majority of households in both the free- and half-price sachet arms (89%) received vouchers in all of the weeks in which the intervention questionnaire was administered. Four households (7%) did not receive vouchers on one of the weeks and two (4%) did not receive vouchers on 2 weeks. One voucher was supplied per child per week, and they were valid for 3 weeks. Overall, the intervention households redeemed an average of 0.88 vouchers/child/week (range = 0.25–1.06 vouchers). This was higher for households in the free-sachet arm (average 0.94 vouchers, range = 0.75–1.06) than those in the half-price sachet arm (average 0.82 vouchers, range = 0.25–1). In the free-sachet arm, redemption rates increased in weeks 2 and 3 and then remained stable (Figure 2

). In the half-price sachet arm, redemption rates decreased slightly after week 1 and continued an overall downward trend over the entire intervention period. One household reported selling their vouchers in a single week and eight reported giving vouchers to other households; seven had given vouchers away once, and one had given them away twice.

In 30.4% of household-weeks, primary caregivers reported consuming sachet water purchased with intervention vouchers. In 80.4% of household-weeks, other household members, including children not enrolled in the intervention (i.e., those ineligible based on their age) and primary caregivers, consumed some of the sachet water obtained with vouchers, and the percentage increased from 62.2% of households in week 1 to 97.7% of households in week 12.

During the first 4 weeks, participants gave 63.8% of the used packaging, equivalent to 102/160 household-weeks, to the intervention team. Those who refused were selling used sachet packaging to waste sub-collectors. After week 4, participants allowed the intervention team to observe used packaging in 90.3% (187/207) of household-weeks. After week 4, an average of 46.7 packages (median = 53; range = 1–117) were observed on each household-week.

Across the intervention period, households reported that they had unused water sachets stored in their home in 42.8% of household-weeks (157/367). The intervention team observed stored water sachets in only 54.8% of those household-weeks (86/157), because interviews were generally conducted outside the participant's residence. On the basis of the direct observation results, households had an average of 12.5 and a median of 8 (range = 1–116) stored sachets. Overall, unused sachets were directly observed at least once during the intervention in 69% of households (37/54) and never observed in 31.5% of households (17/54). Stored sachets were never observed in 29.6% of households (8/27) in the free-sachet arm and 33.3% of households in the subsidized arm (9/27).

The focus group discussions revealed a generally positive response from households in both the half-price and free-sachet arms. All participants reported that their children routinely drank the sachet water, most reported that they also used it to prepare food for their children, and many reported that other household members also drank the sachet water. Sachet water was consumed either directly from the container or from a separate container, such as a bottle or a cup.

### Monitoring questionnaires.

**Figure 3.**
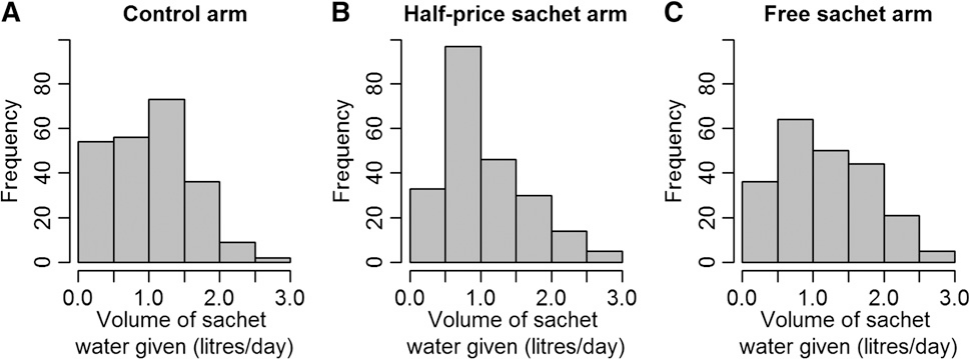
Distribution of daily sachet water volume given to children in the (**A**) control, (**B**) half-price sachet, and (**C**) free-sachet arms. All monitoring weeks are included, giving multiple observations per child.

Overall, 70 children (82%) exclusively consumed sachet water in all 12 monitored weeks; 56 children (69%) exclusively consumed sachet water at baseline and during all monitored weeks. Data were missing for five children. One child in the control arm did not drink sachet water exclusively as the volume (L/day) of sachet water was smaller than the total volume of water consumed in all monitoring weeks. The mean percentage of weeks when only sachet water was used was 91.81%. In the half-price and free-sachet arms, all children consumed sachet water exclusively in at least 75% of the monitoring weeks. The mean percentage of weeks where sachet water was used exclusively was slightly higher in the intervention arms than the control arm, 96.12% for the half-price arm and 96.18% for the free-sachet arm compared with 91.81% in the control arm. The mean volume of sachet water given to each child was 1.28 L/day (range = 0–3 L/day) overall; 1.23 L/day in the control arm (range = 0–2.75 L/day), 1.23 L/day in the half-price sachet arm (range = 0–3 L/day), and 1.39 L/day in the free-sachet arm (range = 0–3 L/day). Figure 3

shows the distribution of sachet water volumes given to children in the three study arms. The distribution of daily sachet water volumes given to the children was significantly different in the half-price and both the control (*P* = 0.005) and free-sachet (*P* < 0.001) arms, but the distributions in the control and free-sachet arms did not differ from one another (*P* = 0.08).

Table 3 shows unadjusted and adjusted coefficients from linear mixed-effects regression models of sachet water volume (L/day) given to children in the three study arms. Age as a categorical variable (3-month age groups), baseline total liquid consumption (liters), and gender were included in the adjusted model. Breastfeeding status was excluded due to colinearity with the child's age. After adjusting for the effects of age, baseline total liquid consumption, and gender, children in the free-sachet arm were given significantly larger volumes of sachet water than children in the control arm (coefficient = 0.27, *P* = 0.03), but the volume of sachet water given to children in the half-price sachet and control arms was not significantly different (coefficient = 0.07, *P* = 0.55).

Logistic regression analysis of exclusive sachet water use (throughout all eight monitoring weeks) versus nonexclusive sachet water use indicated that the covariates age (coefficient = −0.02, *P* = 0.61); gender (coefficient = 0.32, *P* = 0.58); breastfeeding status (coefficient = −0.52, *P* = 0.39); and intervention arm (free-sachet coefficient = −0.09, *P* = 0.90; half-price sachet coefficient = −2.8 × 10^−17^, *P* = 1.0) were not significantly associated with this outcome.

Primary caregivers reported consuming an average of 2.72 L/day of sachet water (median = 2.14 L/day; range = 0–10.71 L/day). The average daily sachet water consumption by primary caregivers was 2.81 L in the control arm (median = 2.50 L, range = 0.71–7.50 L); 2.45 L in the half-price sachet arm (median = 2.14 L, range = 0–0.71 L); and 2.91 L in the free-sachet arm (median = 2.50 L, range = 0.36–8.57 L). Overall, household members including children, primary caregivers, and other residents, consumed an average of 1.84 L/day of sachet water (median = 1.61 L, range = 0–18.75 L).

## DISCUSSION

An increasing proportion of households in west African countries, including Ghana, rely on sachet water as their main source of drinking water.^1^ Economic policies on sales taxes, plastics excise duties, and subsidies impact consumption of sachet water because they affect the price. Robust evidence of the impact of sachet water price on consumption and ultimately on child health, and on the cost-effectiveness of subsidized sachet water, could inform national drinking water policies. This study demonstrated the feasibility of a voucher system to provide subsidized sachet water to low-income households in Accra. The intervention resulted in a statistically significant increase in the volume of sachet water consumed. These results provide information essential for designing and conducting a subsequent, large-scale trial of the impact of subsidized sachet water on child health.

Most caregivers (81%) in these low-income households, even those in the control arm, consistently gave only sachet water to young children during the 12-week follow-up period. There was a high level of acceptance of sachet water as a source of safe drinking water. This finding adds to the understanding of sachet consumption patterns, since existing data on sachet water consumption^3^,^4^ are based on cross-sectional, household-level studies. The published literature on household water treatment and quantitative assessment microbial risk suggest consistent, correct use of sachet water must be maintained to protect against diarrheal disease.^17^,^18^ Exclusive use of treated water may also be necessary if other sources are contaminated.^19^ There is evidence that packaged water may be less prone to fecal contamination at the point of consumption than other widely available alternatives, including piped water.^8^ Therefore, maintaining exclusive, consistent, and correct use of packaged water in low- and middle-income countries could potentially have health benefits. Were these concepts of correct, exclusive, and sustained use from household water treatment studies to be applied to packaged water, correct use of packaged water could include avoidance of unregulated brands and appropriate handling within the home. This is because recent evidence from Sierra Leone suggests that both household storage and contamination of exterior packaging surfaces may contribute to microbial risk.^2^ It could also include appropriate plastic waste management. Although 2010 census data indicated low household sachet consumption in two of the four study EAs, nearly all households were using sachets in 2014–2015. This increase is consistent with previously reported upward trends and volatility in sachet water consumption in Accra^4^ and highlights the importance of consulting multiple sources of information to identify low-sachet-use communities before beginning a larger trial.

The use of vouchers to subsidize sachet water in these low-income households was feasible. The households received the majority of the vouchers to which they were entitled, and most vouchers were redeemed. An average of 0.88 vouchers/child/week were redeemed; one voucher/child/week was provided. The overall voucher redemption rates were higher in the free-sachet arm than in the half-price arm, but the small sample size in this feasibility trial prevented assessing the significance of this difference. A proportion of the subsidized sachets were being used as drinking water by primary caregivers and other household members. In addition, a small number of vouchers were sold or given away to other households. Caregivers consumed an average of 2.72 L/day of sachet water and other householders, including children, consumed an average of 1.84 L/day. Total water consumption data were not collected for caregivers or other householders, but based on an average fluid intake recommendation of 1–2.4 L/adult/day at normal temperatures, 2.8–3.4 L/adult/day at 32°C,^11^ and 2.2 L/day for women 19–50 years of age in temperate climates,^12^ we estimate that sachet water already provides a large proportion of the total water intake for adults in the study households.

There is evidence to suggest that reducing the price of sachet water would increase consumption by young children, despite widespread sachet water consumption at baseline. After adjusting for potential confounders, regression analysis suggested that children in the free-sachet arm were given 270 mL/day more sachet water than children in the control arm. This was statistically significant despite the small sample size, which was not intended to be large enough to detect such differences. Sachet water obtained locally was found to be free of contamination at the outset of the study, and was consumed consistently and exclusively by the children in this trial. Were such consumption patterns to lead to improvements in child health, then this might justify investments to make sachet water more affordable (e.g., via taxation relief or targeted subsidy), increase its safety and reduce its environmental impacts (e.g., via regulation and plastic waste management including deposit-refund systems^20^).

This small-scale study has some significant limitations. Households and the senior trial management team were not blinded to allocation to the intervention arms. Evidence from field studies^14^,^21^ and systematic reviews^22^ of household water treatment suggests that lack of blinding substantially biases self-reported health outcomes. Future work aims to address this via the evaluation of noninvasive, objective measures relating to child diarrhea, for example, weight faltering, pulse, and temperature. It is possible that the provision of free or subsidized sachet water to intervention arm households could have biased the reported sachet consumption from control-arm households, despite the use of systematic sampling to geographically separate the participating households. For example, the number of households reporting that they treated water exceeded the number with treated water available at unannounced visits in studies of home treatment by filtration^23^ and boiling.^24^ There are three steps leading to sachet consumption by young children: successful redemption of vouchers, household acquisition of sachets for consumption, and consumption of sachet water by young children. In any future study, examination of voucher redemption patterns and direct observations of sachets and used sachet packaging stored in the home could be used to mitigate against potential bias in self-reported behaviors regarding the first two steps. Mitigating against biased self-reports of the third step—sachet consumption by children—is more problematic, and would require qualitative methods such as participant observation. Despite this, the patterns of self-reported fluid consumption by children in our study are consistent with biological requirements,^12^ increased with age and decreased with breastfeeding status (Table 3). The direct observation of unused sachets in 68.5% and used sachet packaging in > 90% of households strengthen the evidence for sachet consumption, although not specifically consumption by children.

More generally, this feasibility study was designed with a small sample size and was not powered to detect differences in sachet consumption patterns between arms. A policy change regarding sachet taxation would be unlikely to incorporate an associated educational message, so these findings may overestimate the responsiveness of sachet demand to such a policy-related price reduction. Another limitation was the very high baseline use of sachet water, which was greater than 80%, much higher than reported in previous studies, and severely limited the potential impact of the intervention on water consumption. In a future larger-scale study, this issue could be addressed via an initial rapid appraisal survey to screen out areas of high baseline sachet use. While sachet samples tested here were negative for indicator bacteria, further steps could be taken to protect against sachet contamination in a follow-up trial. For example, there would be scope to cross-reference sachet brands on sale to regulators' databases of registered brands.

The reported use of sachets for drinking by households and children was high at baseline (Tables 1 and 2), contrary to census microdata suggesting otherwise. Elsewhere in Ghana, particularly in rural areas where sachet use is likely to be lower than it is in Accra, provision of vouchers for free or subsidized sachet water could have a much more pronounced effect on water consumption patterns and would require more careful consideration of supply chain and waste management issues. Further research will focus on areas of Greater Accra with low baseline levels of sachet water use, where increased access to sachet water has greater potential to improve health. In comparison, sachet water was not used for cooking: sources such as tanker trucks or public standpipes were more commonly reported as the main source of water for cooking, suggesting that sachet water is preferred for drinking due to convenience or an assumed higher quality when compared with other sources. The sachet water industry and consumption patterns are generally better described in Ghana than in other west African countries, making it unclear how far these findings are generalizable to other countries where water sachets are widely used. However, a recent systematic review^25^ provides evidence that microbial compliance among packaged waters is generally high in low- and middle-income countries.

## CONCLUSION

Baseline and control-arm data suggest that most young children in two low-income neighborhoods of Accra consistently and exclusively consumed sachets over this 12-week period. Although household-level consumption of sachet water has been previously described, intra-household consumption has not. This study provides the first estimates of individual-level sachet consumption. Vouchers for free or subsidized sachet water were redeemed by households that received them and were accepted by participating shopkeepers. Some of this sachet water was consumed by adults and other household members. This feasibility study showed that sachet water consumption increased among children in households receiving vouchers. A large-scale trial of subsidized sachet water targeted at communities with lower baseline sachet consumption could provide greater insight into changes in household consumption in responses to sachet price changes and the potential impact on child health. The data obtained could inform policy affecting the affordability of sachet water and toward this growing industry in general.

## Figures and Tables

**Table 1:** Baseline characteristics of households and children enrolled in the intervention trial

Household characteristics		Control	Half price	Free*	Total
Drinking water (main source)	Sachet	24 (92.31%)	22 (81.48%)	23 (88.46%)	69 (87.34%)
	Piped to neighbor	2 (7.69%)	2 (7.41%)	1 (3.85%)	5 (6.33%)
	Public (tap/standpipe)	0	2 (7.41%)	1 (3.85%)	3 (3.80%)
	Piped into dwelling	0	0	1 (3.85%)	1 (1.27%)
	Piped to compound, yard, or plot	0	1 (3.70%)	0	1 (1.27%)
Cooking water (main source)	Piped to neighbor	5 (19.23%)	6 (22.22%)	8 (30.77%)	19 (24.05%)
	Public (tap/standpipe)	9 (34.62%)	8 (29.63%)	3 (11.54%)	20 (25.32%)
	Piped into dwelling	1 (3.85%)	4 (14.81%)	3 (11.54%)	8 (10.13%)
	Piped to compound, yard or plot	5 (19.23%)	1 (3.70%)	1 (3.85%)	7 (8.86%)
	Tube well/borehole	1 (3.85%)	0	0	1 (1.27%)
	Tanker truck	5 (19.23%)	8 (29.63%)	11 (42.31%)	24 (30.28%)
Storage of drinking water	Covered container	2 (7.69%)	4 (14.81%)	3 (11.54%)	9 (11.39%)
	Sachet	22 (84.62%)	21 (77.78%)	21 (80.77%)	64 (81.01%)
	Do not store at home	2 (7.69%)	2 (7.41%)	2 (7.69%)	6 (7.59%)
Child characteristics					
Gender	Male	15 (51.72%)	12 (42.86%)	12 (44.44%)	39 (46.43%)
	Female	14 (48.28%)	16 (57.14%)	15 (55.56%)	45 (53.57%)
Mean age (months)		19.97	19.9	19.37	19.75
Mean volume of water consumed (L/day)		0.87	0.82	0.97	0.90

* Baseline data are missing for one household.

**Table 2:** Univariable linear regression model of baseline total liquid consumption in L/day of water and other liquids on the day prior to the baseline survey (*N* = 85)

Variable	Category	Coefficient (95% CI)	*P* value	AIC
Age (months)		0.038 (0.02 to 0.06)	< 0.001	191.80
Breastfeeding status	Not breastfed	–	–	–
	Breastfed	−0.85 (−1.16 to −0.53)	< 0.001	182.24
Gender	Female	–	–	–
	Male	0.12 (0.24 to 0.47)	0.51	205.53

AIC = Akaike information criterion; CI = confidence interval.

**Table 3:** Unadjusted and adjusted coefficients from linear mixed-effects regression models of volume of sachet water (L/day) given to children. Repeated measures in each child are accounted for using a panel specification (*N* = 651)

Variable	Category	Unadjusted coefficient (95% CI)	*P* value	Adjusted coefficient (95% CI)	*P* value
Intervention arm	Constant	1.23 (1.03 to 1.42)	< 0.001	0.48 (0.13 to 0.82)	0.006
	Control	–	–	Reference group	
	Half-price sachet	−0.004 (−0.28 to 0.27)	0.98	0.07 (−0.17 to 0.31)	0.55
	Free sachet	0.19 (−0.09 to 0.47)	0.19	0.27 (0.02 to 0.51)	0.03
Age group (months)	6–8	–	–	Reference group	
	9–11	–	–	0.14 (−0.09 to 0.36)	0.24
	12–14	–	–	0.11 (−0.20 to 0.42)	0.49
	15–17	–	–	0.35 (0.02 to 0.68)	0.04
	18–20	–	–	0.51 (0.17 to 0.85)	0.003
	21–23	–	–	0.62 (0.27 to 0.97)	0.001
	24–26	–	–	0.61 (0.23 to 0.99)	0.002
	27–29	–	–	0.61 (0.23 to 0.99)	0.002
	30–32	–	–	0.56 (0.19 to 0.94)	0.003
	33–35	–	–	0.62 (0.25 to 0.99)	0.001
	36–38	–	–	0.36 (−0.09 to 0.81)	0.12
Baseline liquid consumption	(per 1 L change)	–	–	0.20 (0.06 to 0.34)	0.004
Gender	Female	–	–	Reference group	
	Male	–	–	0.06 (−0.14 to 0.26)	0.55

CI = confidence interval.
